# Inequality in health opportunities in Indonesia: long-term influence of early-life circumstances on health

**DOI:** 10.1186/s12889-022-13714-8

**Published:** 2022-07-12

**Authors:** Toshiaki Aizawa

**Affiliations:** Hokkaido University, North 9 West 7, Kita-ku, Sapporo, Hokkaido, 060-0809 Japan

**Keywords:** Equality of opportunity, Non-communicable diseases, Indonesia, I14, I18

## Abstract

**Background:**

This study explores inequality of opportunity in terms of the health of adult Indonesian people, associated with household and parental circumstances in childhood and adolescence.

**Methods:**

Exploiting the longitudinal nature of the Indonesian Family Life Survey, this study measures inequalities relating to being underweight, overweight, hypertensive and diabetic across adult Indonesians aged between 20 and 35 through the dissimilarity index. This study explores their determinants by decomposing the observed inequality levels into contributing factors. Moreover, this study sheds light on the underlying mechanisms through which early-life circumstances influence the health of grown-up respondents, by estimating the intermediate effects of early-life circumstances on current lifestyles.

**Results:**

For all health conditions, health risks are unequally distributed (all *p*<0.01). Demographic factors and parental health are major contributors to inequalities relating to being underweight, overweight and hypertensive. Family structure and parental occupation are major contributors to inequality in diabetes. The greater part of this inequality is explained by the indirect pathways through which early-life circumstances mediate current diet and exercise habits.

**Conclusions:**

The results suggest that such interventions that compensate for disadvantaged early-life circumstances would be essential in reducing future health risks and mitigating health inequality.

**Supplementary Information:**

The online version contains supplementary material available at (10.1186/s12889-022-13714-8).

## Introduction

The Sustainable Development Goals (SDGs), adopted by all United Nations member states in 2015, call for action to ensure healthy lives and promote well-being for people of all ages [1]. Addressing health inequality is essential for sustainable social and economic development. From the viewpoint of responsibility-sensitive egalitarianism [2–6], not all inequalities are ethically objectionable, and two types of inequality can be defined. One is legitimate inequality, in which differences in outcomes are attributable to individual responsibility, and the other is unjustifiable inequality, in which outcome differences are beyond individual control. A typical example of the former is differences in body mass across individuals with different lifestyles, caused, for instance, by dietary choices and daily exercise habits. Such inequality is considered morally ‘fair’, because any differences in consequential health outcomes are the result of an individual’s freedom of choice. On the other hand, unjustifiable inequality is typically exemplified by different mortality rates across different backgrounds, such as genetics, parental social class and race. The inequality of opportunity is one such unjustifiable form of inequality, since causes are beyond the scope of individual control.

In the seminal framework of inequality of opportunity [6], in which every factor influencing individual attainment can be classed as either an effort factor or a circumstance factor. Effort factors are those for which individuals are held partially responsible, while circumstance factors are those that are beyond an individual’s control and are sources of unjustifiable inequality. In the context of health, effort factors typically include lifestyle choices such as diet, smoking, alcohol consumption, exercise and so forth, and disparities that arise unavoidably are typically considered neutral. Circumstances, on the other hand, are typified by race, family socioeconomic and biological background and so on. Effort factors are not necessarily independent of circumstance factors, as some of the former can be more or less influenced by some of the latter [6]. A typical example is that smoking behaviours are more or less affected by parents’ smoking behaviours and socio-economic status (SES). Such effects are assumed to be part of circumstances in the Roemer framework [6].

This study explores the inequality of opportunity in health among people aged between 20 and 35 in Indonesia, in relation to circumstances in their childhood and adolescence. In particular, it focuses on the risk factors for degenerative diseases related to non-communicable chronic diseases such as heart disease, cancers and stroke. As 15 is the age of majority in Indonesia [7], i.e. the recognised threshold of adulthood^1^, we treat circumstantial environments in which individuals live in their childhood and adolescence as factors beyond their responsibility. In other words, this study assumes that the household and parental SES when individuals were children or adolescents aged below 15 are unjustifiable causes of any inequality. From the viewpoint of children, they obviously cannot choose their parents, and therefore the demographic factors and SES of their parents in their childhood and adolescence are beyond their responsibility or control. On the other hand, it seems rather reasonable to assume that, after they reach adulthood, they should be held responsible for the lifestyle they choose. This dynamic analysis is made possible thanks to the large-scale longitudinal Indonesian dataset that has been collected over 20 years.

One of the key features of this study is its use of biomarkers as objective health measurements. Herein, we use the body mass index (BMI), systolic and diastolic blood pressure and glycosylated haemoglobin (HbA1c) as health outcome variables. They objectively reflect an individual’s health and are clinically proven to be reliable [8].

Indonesia has the largest population in South-east Asia, and the fourth largest in the world. During the past two decades, the nation has enjoyed high economic growth, but at the same time, rising health risks inherent in the growth of chronic conditions have been observed [9–11]. Certainly, economic growth is without doubt important for low- and middle-income countries, in that it provides wider opportunities for better health. However, apart from appropriate social policies to ensure reasonable fairness in the way the benefits of economic growth are distributed, it brings little benefit to health equity [12]. A better understanding of health inequality could provide important guidance for policymakers, in order to tackle increasing health risks and mitigate objectionable differences in health for the further social and economic development of the country.

## Data

### The Indonesian Family Life Survey (IFLS)

The Indonesian Family Life Survey (IFLS) is a large-scale, ongoing longitudinal survey and was designed and implemented by the RAND Corporation, launched initially in 1993/94. It currently has five waves, the latest of which was completed in 2014. Hence it has a 21-year gap between the first wave and the last wave. The survey questions are very extensive, covering a household’s economy, education, employment and a wide range of health conditions. The sample is representative of people living in 13 of the 27 provinces in the country, where about 83 per cent of the total population resides. Four provinces on Sumatra (North Sumatra, West Sumatra, South Sumatra and Lampung), all five of the Javanese provinces (DKI Jakarta, West Java, Central Java, DI Yogyakarta and East Java) and four provinces covering the remaining major island groups (Bali, West Nusa Tenggara, South Kalimantan and South Sulawesi) are included [13]. The choice of the province was based on cost considerations for survey implementation, without compromising on the coverage of socio-economic and ethnic diversity [13]. To make the sample national-representative, sample weights are used. The high re-contact rates of each wave are one of the strengths of the IFLS. For example, the re-contact rate in the latest wave among households interviewed in the first wave was reported at 92.0%. Among those households interviewed in the first wave, 86.9% were involved in all five waves, thereby helping to lessen the potential risk of bias owing to non-random attrition. In fact, its re-contact rates are as high as – or higher than – most longitudinal surveys available in the United States and Europe [13]. This study focuses on grown-up children aged between 20 and 35. This young age category is selected because respondents who had not reached adulthood in the first wave are at most 35 years old. Also, respondents who were surveyed in the first wave should be at least 20 years old when they were surveyed again in the last wave. We do not consider the respondents who are over 35 years of age in the last wave because we cannot access the early-life circumstances by exploiting the longitudinal nature of the IFLS.

### Outcome variable

The health biomarkers in this study are BMI, systolic and diastolic blood pressure and HbA1c. Information on these biomarkers in the IFLS was based on actual measurements taken by health workers (nurses), who had received special training, or via laboratory-based blood examinations, thereby substantially enhancing the credibility of the results. First, BMI, which was calculated from the height and weight of the respondents, is defined as an individual’s weight divided by their height squared and expressed internationally in units of *kg*/*m*^2^. People are diagnosed as being underweight if their BMI is below 18.5, and overweight if their BMI is over 25.0.

Second, systolic and diastolic blood pressures were measured three times by nurses. In this study, the average of these three measurements is used to assess if people have hypertension. This paper follows the WHO definition of hypertension, namely an average systolic blood pressure that is equal to or greater than 140 millimetres of mercury (*mmHg*) or an average diastolic blood pressure which is equal to or greater than 90 *mmHg* [14]. High blood pressure is one of the most well-known causes of life-threatening complications such as heart attack, stroke, kidney failure and premature mortality [14]. Finally, for HbA1c, a finger prick was taken and blood spots collected, following which the dried blood spots were analysed in medical laboratories. A high level of HbA1c equal to or greater than 6.5% is indicative of diabetes [15], which is equivalent to 7.7 *mmol*/*l* and 47.5 *mmol*/*mol*. HbA1c is a measure of glucose metabolism and is used to diagnose diabetes mellitus [16]. These non-communicable diseases are not independent of the recent global Covid-19 pandemic, as they increase the risk of becoming severely ill from Covid-19 [17, 18]. Those who take medication for diabetes/hypertension are also categorised as being diabetic/hypertensive regardless of their measured HbA1c values or blood pressure.

### Circumstance variables

**Fig. 1:**
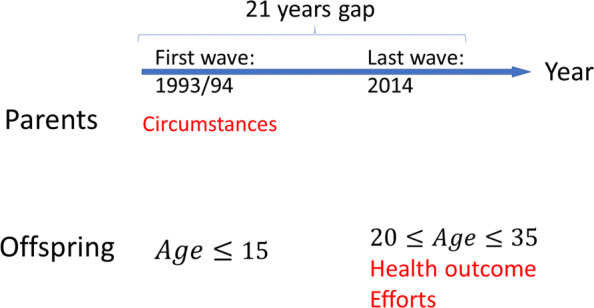
Twenty-one year gap in the IFLS

This study assumes that the respondents’ circumstances before they reach adulthood were beyond their control and responsibility. As 15 is the age of majority in Indonesia [7], circumstances when the respondents were below this age are considered unfair sources of inequality. This study exploits the panel structure of the IFLS to obtain them, i.e. the 21-year gap between the first wave (completed in 1993/1994) and the last wave (completed in 2014) (Fig. 1). Household and parental SES in 1993/1994 may well be recognised as circumstances from the viewpoint of their adult offspring surveyed in 2014, which was made possible thanks to the high re-contact rates in the IFLS.

This study considers multidimensional circumstances as found in the real world. From the children’s perspective, parental educational background, occupation and location of domicile can be regarded as important factors, as well as household income or wealth. Consideration of the multidimensionality of circumstances allows us to explore in-depth health inequality that cannot be captured by just a single index [19]. Taking account of various sources of inequality is especially important, as there is consensus that health inequality determinants are wide-ranging and composite factors, including affluence, education, occupation and so on, which together represent various aspects of SES better than any of these elements alone do [20].

This study considers the nine-dimensional circumstance categories listed in Table 1, following previous studies on child and adult health inequality [21–24]. Circumstance variables are categorised herein as (1) demography, (2) location (3) family structure, (4) parental health, (5) parental education, (6) living standards, (7) housing, (8) parental occupation and (9) healthcare access. For demography, we consider the respondents’ sex, ethnicity (Javanese/non-Javanese) and religion (Islam/Hindu/Catholic/Others). The second category, location, considers the province in which respondents lived when they were below 14 years old and whether they resided in urban or rural areas. In general, in developing countries, children in urban areas have a better nutritional status and a lower risk of death [25, 26]. A third category, family structure, considers whether mothers had teenage pregnancies, whether parents were not divorced at age 12 and whether respondents lived with biological mothers and fathers at age 12. Furthermore, the family structure also includes family size in households, because the number of siblings in a household is a significant factor in household resource allocations [27]. The negative effects of family size on child health outcomes are reported in multiple countries [28–31].

**Table 1:** List of circumstance categories

Category	Variables
Demography	Sex, religion, ethnicity
Location	Provinces, urban
Family structure	Mother’s teenage pregnancy, parental divorce ^*†*^, living with biological mother and farther ^*†*^, family size
Parental health	Paternal BMI, maternal BMI, paternal height, maternal hight
Parental education	Paternal education years, maternal education years
Living standards	wealth ^∗^, number of books ^*†*^, hunger experiment ^*†*^,
Housing	Wall, roof, floor, electricity access, clean water access, clean toilet, Sanitary conditions
Parental occupation	Self-employment, government worker, private sector worker, family business worker, industry types (primary/secondary/service)
Healthcare access	Knowledge of local public hospitals, private hospitals, public health centres (puskesmas), private clinics, private physicians, nurses/paramedics/midwife practitioners.

Note: ^*†*^ This is when respondents were 12 years old. ^∗^The logarithmic family-size adjusted amount is used

We also consider the health of parents when their offspring were below 14 years old. Intergenerational transmission of health, which is a result of both genetic inheritance and family environment, is found in many countries, and a significant correlation between parental and child health persists even after children have grown up [32–34]. For example, the existence of the intergenerational transmission of BMI is recognised internationally, and parental over- and under-nutrition is a highly significant predictor of BMI in their offspring [35–39]. The fundamental reason not to use contemporaneous parents’ BMI is that they are not necessarily beyond the control of grown-up offspring. It is quite likely that grown-up sons/daughters encourage their parents to try to participate in physical activities, or give advice on leading a healthy lifestyle. It is also likely that grown-up children play important roles in the choice of daily meals for their parents. In these cases, parental BMI may not be categorically viewed as beyond control from the viewpoint of offspring. On the other hand, before children mature into adulthood, they are far less likely to influence their parents’ BMI. In addition, we consider parental heights as circumstances, which are known to be one of the significant factors for child health [39, 40].

For parental education, we take account of the maternal and paternal education years as improved infant health is reported among children with educated mothers [41–43]. In addition, malnourished women tend to deliver smaller babies and are less successful at breastfeeding their children [44, 45]. For living standards, we include per capita household wealth as a proxy, reflecting living standards when respondents were in childhood or adolescence. The advantage of using wealth over income is that the former, as a stock of income, is suitable as an indicator reflecting the long-term living standards of households [9]. Wealth is defined as the aggregated total value of the various assets commonly found in typical Indonesian households of which details are available in the Additional file 1: Appendix. In addition, the living standard category includes the number of books in a house when respondents were aged 12 and whether respondents experienced hunger at the same age. Household affluence is one of the important socio-economic factors that create inequality in health [46, 47].

The seventh category, housing, considers whether the house in which respondents lived had finished walls, roofs and floors, whether the house had electricity access and clean toilets and whether the household disposed of its garbage in trash cans collected by a sanitation service. Housing and sanitary conditions are significant determinants of child health [48–53]. For parental occupation, we consider the type of paternal and maternal occupation: self-employed worker, government worker, private-sector worker or family business worker. The occupation category also takes into account industry types: primary, secondary or service industries. The significant relationship between child health and parental employment status is reported in previous studies [54–56].

The last category, healthcare access, considers the knowledge of household heads in terms of local healthcare facilities: public hospitals, private hospitals, public health centres (*puskesmas*), private clinics, private physicians and nurses/paramedics/midwives. This study considers the knowledge of healthcare facilities because the IFLS does not have detailed information about the actual utilisation of them. For respondents who grew up in single-parent families, as their parental information regarding education, health and occupation were partially available, we impute the missing information from that of single foster parents.

### Descriptive statistics

After dropping the outliers, which are defined as the top 0.1% or bottom 0.1% of BMI of respondents, parental BMI and parental heights, from the sample and deleting observations with missing values, the working sample sizes are 5,523 for the conditions of being underweight and overweight, 5,481 for hypertension and 1,148 for diabetes. As dried blood spots were sampled from the randomly chosen household sub-sample, there are fewer observations for HbA1c than for the other biomarkers. We fail to reject the equalities of the health outcome distributions of the complete observations and that of the deleted incomplete observations with parental information missing. Also, we did not find significant relationships between attrition and health. The descriptive statistics shown in the Additional file 1: Appendix indicate that 11% and 30% of the respondents in the sample are underweight and overweight, respectively, and that 11% are hypertensive and 4% diabetic.

## Method

### The measurement of inequality of opportunity

In Roemer’s seminal framework [57], all factors influencing an outcome variable are sorted into effort factors, *E*={*E*_1_,…,*E*_*m*_}, and circumstance factors, *C*=(*C*_1_,…,*C*_*q*_). It assumes that efforts are also correlated with circumstances and that the outcome has a general function *Y*=*g*(*C*,*E*,*ε*)=*g*(*C*,*E*(*C*,*v*),*ε*), where *v* and *ε* are unobserved error terms; specifically, *v* reflects the random variations in effort that are independent of *C*, and *ε* captures random variation in outcomes that are independent of *C* and *E*. Any variation in *Y*, driven by the distribution of circumstances, is regarded as the inequality of opportunity. When we employ the reduced-form probability model for health risks as *p*=*Pr*(*Y*=1)=*f*(*C*), the inequality of opportunity can be measured by inequality in the predicted probabilities, as the reduced-form probability model reflects only – and fully – the unjustifiable determinants of inequality. We use the logistic regression to estimate the predicted probabilities.

We quantify the inequality of opportunities in health via the dissimilarity index (D-index), which is based on a comparison of the predicted probabilities of different people with mean probabilities, and it measures how predicted probabilities are dissimilar according to circumstances [58]. The D-index has been used to measure inequality associated with circumstantial factors [59–61]. The D-index is calculated as

1 $$\begin{array}{@{}rcl@{}} D(\widehat{p_{i}})=\frac{1}{2\bar{p}}\sum_{i=1}^{n}\frac{1}{n}|\widehat{p_{i}}-\bar{p}|, \end{array} $$

where *n* is the sample size, $\widehat {p_{i}}$ is a predicted probability of *Y*=1 for individual *i* and $\bar {p}=\frac {1}{n}\sum _{i=1}^{n}\widehat {p_{i}}$ is mean predicted probabilities. The latter part of Eq. (1), $\sum _{i=1}^{n}\frac {1}{n}|\widehat {p_{i}}-\bar {p}|$, indicates the average absolute disparity of individual predicted probability from the average. Standardising this average absolute disparity by dividing it by $2\bar {p}$, the D-index measures the relative degree of dissimilarity level, ranging from 0 to 1, with 0 indicating an equal chance of having *Y*=1 across people with difference circumstances. Quantitative interpretation is also possible, in that the D-index measures the relative proportion of available opportunities that needs to be re-distributed from better-off to worse-off groups, in order to achieve equality of opportunity.

### Shapley decomposition of the d-index

Understanding the determinants of inequality is of great interest to policymakers and public health specialists seeking to identify the determinants of inequality and possible paths for intervention, in order to mitigate inequality. The D-index can be decomposed into its contributory factors by the Shapley decomposition [62], which is built around the expected marginal contributions of each circumstance variable and calculates the marginal impacts of each variable on the relevant inequality.

Our Shapley value decomposition works as follows. We pick one of the circumstance categories, say *C*_1_, and replace its original values with its sample means, i.e. $\overline {C_{1}}$, for all people. Using $\overline {C}_{1}$, we predict the hypothetical probability with the probability model that we estimated, namely $\hat {p}_{-1i}=\hat {f}(\overline {C_{1}},C_{2i},\dots,C_{qi})$, from which we calculate $D(\hat {p}_{-1i})$. We can then quantify the marginal contribution of *C*_1_ to total inequality by taking the difference between the D-index calculated with the originally predicted probability and that calculated with the hypothetical predicted probability, $D(\hat {p}_{i})-D(\hat {p}_{-1i})$. Intuitively, we estimate the contribution of *C*_1_ by subtracting the hypothetical inequality level in which the effect of *C*_1_ is suppressed from the overall observed inequality. In the following step, we replace another variable, say *C*_2_, and calculate its marginal contribution in the same manner. Namely, we calculate $D(\hat {p}_{-1i})-D(\hat {p}_{-1-2i})$, where $\hat {p}_{-1-2i}=\hat {f}(\overline {C_{1}},\overline {C_{2}},C_{3i},\dots,C_{qi})$. We subsequently replace all independent variables with their sample means and calculate how much inequality is marginally reduced when nullifying the effect of each circumstance variable. Considering all the possible orders in the calculation of the marginal contribution of each variable, we obtain a great number of marginal contributions for each variable. Averaging all the possible marginal contributions for each variable yields the average marginal contribution made by each variable. The advantage of the Shapley value decomposition lies in the fact that the result is path-independent, i.e. the order when nullifying each covariate does not affect the final result, and the sum of all contributions corresponds to the value of overall inequality. We use Stata/MP 16.0 for computing the D-index and implementing the Shapely decomposition.

## Results

### Dissimilarity index

**Fig. 2:**
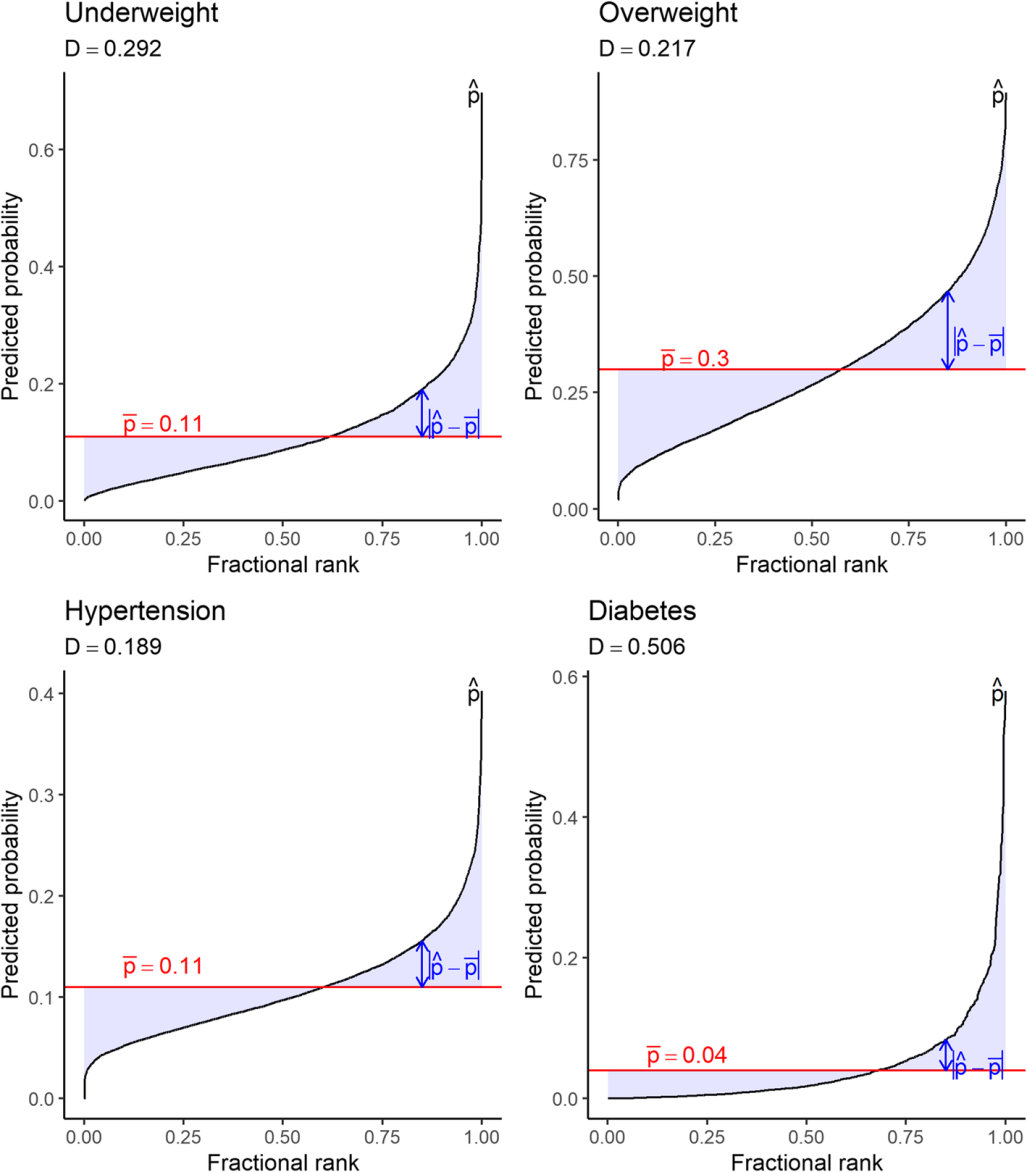
Predicted probabilities for health outcomes. Note: The sorted predicted probabilities are plotted. The shaded areas indicate the difference between the predicted probability and the mean probability

Figure 2 plots the sorted predicted probabilities for being underweight, overweight, hypertensive and diabetic, respectively. The horizontal axis indicates the fractional rank of the probability size – and thus the differences in sample sizes across health outcomes is taken into account. As variations in predicted probabilities are driven solely by the circumstance factors, the difference between individual predicted probabilities reflects an inequality of opportunity. All health outcomes indicate substantial variations in predicted probabilities, with the probabilities of being diabetic exhibiting the largest variation. The shaded area denotes the difference between the predicted probability and the mean probability, indicated by the horizontal line in red. Total shaded areas measure the total absolute disparity of individual predicted probability from the mean probability, from which we calculate the D-index.

Table 2 reports the D-indices of four conditions and their decomposition results. The D-indices show statistically significant values for all four outcomes (*p*<0.01). For underweight, the D-index is 0.292 [95% confidence interval (CI): 0.265-0.318], and the decomposition analysis indicates that the parental health is the largest contributor, showing the value of 0.111 and explaining around 38.1% (=100*0.111/0.292) of the estimated D-index (*p*<0.01). Its large contribution can be attributable to the intergenerational transmission of health from parents. Demographic factors are the second largest contributor at 0.075, explaining 25.9% (=100*0.075/0.292) of the estimated inequality (*p*<0.01). For overweight, its D-index is 0.217 [95% CI: 0.201-0.234]. Similar to the underweight case, parental health and demographic factors are the two largest contributors, explaining 32.0% and 32.4% of the overall D-index, respectively (*p*<0.01). Different from the case of the underweight condition, parental educational background shows a significant association (*p*<0.01), although its relative contribution size is far smaller than other circumstance categories.

**Table 2:** Dissimilarity index

	Estimates	95% CI	Proportion	Estimates	95% CI	Proportion
	**Underweight**			**Overweight**		
Overall						
Dissimilarity index	0.292***	(0.265,0.318)	1.000	0.217***	(0.201,0.234)	1.000
Decomposition						
Demography	0.075***	(0.053,0.097)	0.259	0.07***	(0.056,0.085)	0.324
Location	0.003	(-0.003,0.009)	0.011	0.005***	(0.001,0.009)	0.023
Family structure	0.033***	(0.018,0.047)	0.113	0.025***	(0.018,0.032)	0.116
Parantel health	0.111***	(0.09,0.133)	0.381	0.07***	(0.059,0.081)	0.320
Parental education	0.004	(-0.002,0.011)	0.015	0.004***	(0.002,0.006)	0.018
Living standards	0.008**	(-0.001,0.016)	0.026	0.003***	(0.001,0.006)	0.015
Housing	0.024***	(0.012,0.035)	0.081	0.016***	(0.011,0.022)	0.075
Parantal occupation	0.021***	(0.009,0.033)	0.072	0.013***	(0.008,0.019)	0.062
Healthcare access	0.012***	(0.003,0.022)	0.042	0.01***	(0.006,0.015)	0.047
	**Hypertension**			**Diabetes**		
Overall						
Dissimilarity index	0.189***	(0.163,0.214)	1.000	0.506***	(0.398,0.614)	1.000
Decomposition						
Demography	0.047***	(0.028,0.067)	0.249	0.039	(-0.013,0.091)	0.078
Location	0.003	(-0.004,0.011)	0.018	0.03	(-0.008,0.067)	0.059
Family structure	0.035***	(0.018,0.052)	0.184	0.198***	(0.125,0.27)	0.391
Parantel health	0.045***	(0.026,0.063)	0.236	0.028	(-0.008,0.065)	0.056
Parental education	0.003	(-0.003,0.009)	0.015	0.025	(-0.011,0.06)	0.049
Living standards	0.011**	(0.001,0.02)	0.056	0.037*	(-0.002,0.075)	0.072
Housing	0.02***	(0.006,0.034)	0.107	0.041*	(-0.005,0.087)	0.081
Parantal occupation	0.016***	(0.003,0.028)	0.083	0.073***	(0.023,0.123)	0.144
Healthcare access	0.01*	(-0.001,0.021)	0.051	0.036*	(-0.002,0.074)	0.071

Note: CI=Confidence interval. A 95% confidence interval is calculated by bootstrap with 500 repetitions. * *p*<0.10, ** *p*<0.05, *** *p*<0.01

Third, the D-index for hypertension is the smallest of all four health outcomes at 0.189 [95% CI: 0.163-0.214], but it is still significantly above zero (*p*<0.01). The demographic factor indicates the largest contribution at 0.047 and explains a quarter of the observed inequality (*p*<0.01). Parental BMI is the second largest contributor at 0.045, explaining 23.6% of the inequality (*p*<0.01). Last, for diabetes, its D-index is 0.506 [95% CI: 0.398-0.614], and family structure and parental occupation show the two largest significant contributions (*p*<0.01), explaining 39.1% and 14.4% of total inequality, respectively. Different from the other three health conditions, we do not find that parental health makes a significant contribution to inequality in diabetes.

### Exploration of the intermediate pathway

So far, we have quantified the inequalities of health conditions associated with circumstances in childhood and adolescence. However, the potential mechanisms by which early-life circumstances influence adult health are not yet examined. Next, we explore possible mechanisms by looking at the intermediate influences of early-life circumstances on current lifestyles.

It is quite probable that environments in childhood and adolescence shape children’s health-related behaviours and psychological traits, such as preference. For example, food preference can be influenced in the long term by meals offered when they are very young. First, we estimate the effect of circumstances on effort variables, following which we obtain the predicted effort values, $\hat {E}=\hat {e}(C)$. For continuous effort variables, we use ordinary least squares (OLS) regression to estimate the relationship between circumstances and effort, and for dichotomous effort variables, we use the probit regression model so that the predicted probability ranges between 0 and 1. Estimation results are available upon request from the author. Second, we estimate the D-index, using these predicted effort variables. The estimated D-index may well be recognised as the part of the inequality that is indirectly induced by early-life circumstances through effort. By decomposing the D-index, we explore what pathways are strong drivers of inequality.

A set of our effort variables is composed of the following five categories: 1) Respondent’s education, 2) living standards, 3) food expenditure and choices, 4) exercise and 5) occupation (Table 3). The choice of these effort variables is based on the previous studies on non-communicable diseases in developing countries [63–65] and the epidemiological and public health literature on nutrition [66–68]. The information is obtained from the most recent IFLS in 2014/15, when the respondents have already reached adulthood (Fig. 1). First, the respondent’s educational background is measured by education years. Second, for variables reflecting household living standards, we consider logarithmic family size-adjusted household wealth, whether the household has access to clean water, whether the household owns a clean toilet and whether the household uses safe cooking fuel.

**Table 3:** List of effort variables

Category	Variables
Education	Education years
Living standards	Wealth ^∗^, clean water, clean toilet, safe fuel use
Diets	Food expenditure ^∗^, prepared food ratio, staple food ratio, frequencies of consuming fast foods, soft drinks, fried snacks, and sweet snacks
Exercise	Vigorous exercise, moderate exercise
Occupation	Self-employment, government worker, private sector worker, family business worker, industry types (primary/secondary/service)

Note: ^∗^The logarithmic family-size adjusted amount is used

Third, for measurements of food expenditure and choices, the logarithmic amount of family size-adjusted total expenditure on food is used. In the survey, the reporting of food expenditure information was done according to household unit. This study assumes that household members share expenditure on an equal basis. The proportion of expenditure spent on prepared foods outside the home to total food expenditure is considered in this study, as it captures the different food choices made by the household. Moreover, the analysis also includes the share of food expenditure spent on staple foods such as hulled, uncooked rice, sago/flour, cassava and tapioca, which is important, because developing Asian countries are currently experiencing a nutrition transition from traditional diets to Western diets [69, 70]. As in traditional Indonesian cultures, unprocessed staples have played a major role [11], and so a lower share of staple food could be considered to indicate the lesser importance of traditional cuisine in diet choice. Furthermore, we consider the frequency of eating unhealthy foods, in the form of instant noodles, fast foods, soft drinks, fried snacks and sweet snacks, over a one-week period.

Fourth, exercise includes daily vigorous and moderate physical activities. In the IFLS, vigorous activities are defined as those that make one breathe much harder than normal and may include heavy lifting, digging, ploughing, aerobics, fast cycling and cycling with a load. Moderate activities make one breathe somewhat harder than normal and may include carrying light loads, cycling at a regular pace or mopping the floor. For both vigorous and moderate physical activities, work that takes fewer than ten minutes is not counted as an exercise. The intensity of these exercises is calculated according to time spent and the frequency of the respective activity. The variable is the product of the time they usually spend in a day (1 is given if they did no exercise; 2 is given to less than 30 minutes’ exercise; 3 is given to exercises between 30 minutes and 4 hours; 4 is given if it is more than 4 hours) and the days on which they do this in a week. The lowest value is zero (no exercise in a week) and the highest is 28 (more than 4 hours and 7 days a week). Finally, we consider the respondent’s current occupation – self-employed worker, government worker, private-sector worker or family business worker. Table 4 summarises the results after measuring the proportion of inequality caused solely by the indirect pathway in relation to the overall D-index shown in Table 2. We find that a large proportion of the overall inequality is associated with the indirect pathways by which circumstances influence health via effort. First, for the underweight category, the D-index associated with the indirect pathways is 0.204 [95% CI: 0.176-0.232], which means that 69.9% (=100*0.204/0.292) of the observed total inequality is explained by indirect pathways (*p*<0.01). Looking at the decomposition result, we find that current dietary choice makes the largest contribution at 0.060 [95% CI: 0.042-0.077], which corresponds to 20.5% (=100*0.06/0.292) of the observed inequality, followed by exercise at 0.052 [95% CI: 0.037-0.067], which is 17.8% (=100*0.052/0.292) of the overall inequality.

**Table 4:** Dissimilarity index associated with the indirect pathway

	Estimates	95% CI	Proportion	Estimates	95% CI	Proportion
	**Underweight**			**Overweight**		
Overall						
Dissimilarity index	0.204***	(0.176,0.232)	0.699	0.171***	(0.155,0.187)	0.785
Decomposition						
Education	0.012***	(0.006,0.017)	0.040	0.011***	(0.008,0.013)	0.049
Living standards	0.031***	(0.017,0.045)	0.106	0.029***	(0.023,0.035)	0.133
Diets	0.06***	(0.042,0.077)	0.205	0.04***	(0.034,0.046)	0.185
Exercise	0.052***	(0.037,0.067)	0.178	0.049***	(0.041,0.058)	0.227
Occupation	0.05***	(0.036,0.064)	0.171	0.042***	(0.035,0.048)	0.191
	**Hypertension**			**Diabetes**		
Overall						
Dissimilarity index	0.13***	(0.102,0.159)	0.690	0.308***	(0.186,0.431)	0.610
Decomposition						
Education	0.003	(-0.003,0.01)	0.018	0.018	(-0.007,0.044)	0.036
Living standards	0.018***	(0.004,0.031)	0.093	0.055***	(0.011,0.099)	0.108
Diets	0.047***	(0.03,0.063)	0.247	0.095***	(0.034,0.156)	0.188
Exercise	0.026***	(0.015,0.037)	0.138	0.032*	(-0.004,0.068)	0.063
Occupation	0.037***	(0.023,0.05)	0.194	0.108***	(0.049,0.168)	0.214

Note: CI=Confidence interval. A 95% confidence interval is calculated by bootstrap with 500 repetitions. * *p*<0.10, ** *p*<0.05, *** *p*<0.01

For the overweight category, the D-index related to the indirect pathway is 0.171 [95% CI: 0.155-0.187], and we observe that 78.5% (=100*0.171/0.217) of the observed inequality due to early-life circumstances is explained by the intermediate pathways (*p*<0.01). Exercise, diet and occupational type play significant roles with contributions of 0.049, 0.040 and 0.042, respectively (*p*<0.01). These findings suggest that inequalities related to being underweight and overweight among grown-up offspring are significantly associated with the mechanism through which early-life circumstances influence current diets and exercise habits.

For hypertension, the D-index owing to the indirect pathway is 0.130 [95% CI: 0.102-0.159], thus implying that 69.0% (=100*0.130/0.189) of the observed inequality is explained by the intermediate pathways. All effort variables, except education, make significant contributions (*p*<0.01). The pathway through the diets exhibits the largest value, namely 0.047 [95% CI: 0.030-0.063], and it explains 24.7% of overall inequality, followed by occupation type, which accounts for 19.4% of the observed total inequality. Lastly, for diabetes, the D-index associated with the indirect pathway is 0.308 [95% CI: 0.186-0.431], and we observe that 61.0% (=100*0.308/0.506) of the overall inequality is explained by the indirect pathways. The pathways through diets and occupation type make large contributions, accounting for 18.8% and 21.4% of the total inequality (*p*<0.01). The pathways through education and exercise habits do not make significant contributions to inequality at a 5% level.

## Discussion

Although these estimates do not necessarily reflect a causal relationship between them, the results of this study suggest that such interventions that compensate for disadvantaged early-life circumstances would be essential in reducing future health risks and mitigating health inequality. Introducing or reinforcing policies targeting children from the marginalised households, such as cash transfer programmes, in-kind transfers and voucher programmes, should be considered as instruments to alleviate the adverse effect of circumstances on health [71–74]. The relatively larger contributions made by parental health suggest that addressing parental adult health problems such as excess weight could be beneficial in terms of both improving their health and ameliorating inequality in children’s health in later years. Given the well-established relationship between parental education and child health in developing countries [75–78], enhancing health-related literacy among parents would also be an important step in addressing the vicious cycle of intergenerational health risks.

Even for those circumstances that cannot be amended directly by household interventions, ensuring that improving underprivileged environments does not become an obstacle or a handicap to child development is of paramount importance, too. It is therefore important to weaken the link between such circumstantial characteristics and development, so that every person has equal developmental opportunities regardless of their sex, religion or ethnicity. Promoting health-related literacy at schools, such as via education related to nutrition and physical activity, would help to attenuate the association between early-life circumstances and health in later life, as such education could play a part in preventing disadvantaged early-life circumstances from negatively influencing lifestyle choices in later life. Achieving equality in terms of opportunity, whereby all individuals have access to the same opportunities for better health, regardless of their circumstances, by levelling the playing field would help facilitate Indonesia’s sustainable development.

## Conclusion

This study explored the inequality of opportunity in health among Indonesian people aged between 20 and 35, which can be attributed to their early-life circumstances – for which they should not be held responsible. The study exploited the longitudinal nature of IFLS data and measured inequality in health risks associated with circumstances when respondents were in their childhood and adolescence. We decomposed overall inequality into its determinants, in order to identify the drivers of such inequality.

The results indicate that the distribution of health risks is significantly related to the early-life environment in which the respondents grew up. For the underweight, overweight and hypertension conditions, parental health makes a large contribution to their inequality. Family structure and parental occupation make significant contributions to all health outcomes. Living conditions make significant contributions to inequalities in relation to being underweight, hypertensive and diabetic. Housing environments are significantly related to inequalities in terms of being underweight, overweight and suffering from hypertension. Compared to the other circumstances, the contribution made by the parental educational background is relatively small, but it makes a significant contribution to inequality for being overweight.

This study explored the possible pathways by which early-life circumstances influence health, by estimating the inequality attributable to the indirect pathways through various intermediate lifestyle choices – for which individuals should be held responsible. More than half of the observed inequalities are explained by the intermediate pathways through which early-life circumstances influence adult health via current lifestyle choices and SES. Especially, relatively larger proportions of overall inequality are explained by the indirect pathway via diet choice, exercise habits and current occupational SES.

Last of all, it is worth discussing the limitations of this study. First, in terms of the location of domicile, financial situation and political autonomy differ even at the district level in Indonesia [79], which are expected to affect child health through material resource availability. This study takes account of such effects only at the province level and heterogeneous levels in authority at a district or municipality level may not have been fully captured in the analysis. Second, the D-index employed in this study is designed to capture the variation in an outcome that is associated with the observable variables in the model. This study includes a comprehensive set of household and parental characteristics, but they are just a subset of conceivable circumstantial factors for their offspring. Parental preference, for example, should be potentially regarded as circumstances in the sense that they are not controllable by children. In this sense, the inequality measured by the D-index should be interpreted as the fraction of the entire inequality.

## Supplementary Information

**Additional file 1** Supplementary file.

## Data Availability

The data in this study is publicly available.

## Footnotes

1 Although age of majority in Indonesia varies across legal instruments, this study follows the one used in the criminal code.
