# Supplementary material for: Inequality in health opportunities in Indonesia: long-term influence of early-life circumstances on health
Source: BMC Public Health. 2022 Jul 12;22:1334. doi: 10.1186/s12889-022-13714-8 (PMC9278321; doi:10.1186/s12889-022-13714-8)
Supplement: Supplementary file 1 — Additional file 1 Supplementary file. [file 12889_2022_13714_MOESM1_ESM.pdf]

Supplementary file

A Appendix

A.1 Definition of wealth

Wealth is defined as the aggregated total value of the following various assets commonly found in typical Indonesian households: House and land occupied by a household; Other houses/building (including land); Land (not used for farming); Poultry; Livestock/fish pond; Hard stem plant not used for farming, or a non-farm business; Vehicles (cars, boats, bicycles, motorbikes); Household appliances (radio, tape-recorder, television, fridge, sewing or washing machine, video and CD player, cell phone, etc.); Savings/certificates for deposits/stocks; Receivables; Jewellery; Household furniture and utensils.

A.2 Supplementary results

Table A.1: Descriptive statistics of health outcomes

	count	mean	sd
Underweight	5523	0.11	0.31
Overweight/obese	5523	0.30	0.46
Hypertension	5481	0.11	0.31
Diabetes	1148	0.04	0.19

Table A.2: Descriptive statistics of circumstances

	mean	sd
Paternal BMI in childhood	21.23	2.72
Maternal BMI in childhood	21.87	3.24
Paternal height in childhood	158.81	6.68
Maternal height in childhood	150.06	5.23
Paternal education years	6.96	3.61
Maternal education years	5.91	3.57
ln(Wealth) in childhood	12.63	2.57
Books 11-25	0.15	0.36
Books 26-100	0.06	0.23
Books 101-200	0.01	0.09
Books 200-	0.00	0.06
Had hunger	0.04	0.20
Javanese	0.42	0.49
Male	0.47	0.50
Islam	0.90	0.31
Hindu	0.05	0.22
Catholic	0.01	0.10
Family size in childhood	5.82	1.94
Teenage pregnancy	0.12	0.33
Mother married at age 12	0.95	0.21
Biologicaal mother at age 12	0.97	0.18
Biologicaal father at age 12	0.93	0.26
Electricity	0.65	0.48
Clean water	0.12	0.33
Clean toilet	0.25	0.44
Sanitation	0.14	0.35
Finished wall	0.47	0.50
Finished floor	0.58	0.49
Finished roof	0.92	0.28
Number of rooms	4.60	1.96
Father self-employed worker	0.54	0.50
Mother self-employed worker	0.23	0.42
Mother goverment worker	0.11	0.31
Father goverment worker	0.04	0.21
Mother private sector worker	0.31	0.46
Father private sector worker	0.09	0.29
Mother family worker	0.01	0.08
Father family worker	0.17	0.38
Mother primary industry	0.41	0.49
Father primary industry	0.23	0.42
Mother secondary industry	0.09	0.28
Father secondary industry	0.29	0.45
Mother service industry	0.27	0.44
Father service industry	0.23	0.42
Knowledge of public hospitals	0.66	0.47
Knowledge of private hospitals	0.31	0.46
Knowledge of health centres	0.94	0.24
Knowledge of private clinics	0.13	0.34
Knowledge of private physicians	0.47	0.50
Knowledge of nurses/paramedics/midwife practitioners	0.71	0.45
Urban in childhood	0.40	0.49
North Sumatra	0.09	0.28
West Sumatra	0.05	0.22
South Sumatra	0.06	0.24
Lampung	0.06	0.23
West Java	0.14	0.35
Central Java	0.14	0.35
Yogyakarta	0.04	0.19
East Java	0.12	0.32
Bali	0.06	0.23
West Nusa Tenggara	0.09	0.29
South Kalimantan	0.05	0.22
South Sulawesi	0.05	0.22
Observations	5563	

Table A.3: Descriptive statistics of efforts

	mean	sd
Education years	11.02	3.42
ln(Wealth)	16.50	1.81
ln(food expenditure)	12.90	0.63
Prepared food ratio	0.15	0.19
Staple food ratio	0.18	0.15
Vigorous activity	2.36	5.76
Moderate activity	5.77	7.44
Instant noodle freq.	1.95	1.95
Fast food freq.	0.25	0.78
Soft drink freq.	0.50	1.19
Fried snacks freq.	2.39	2.53
Sweet snacks ferq.	2.13	2.53
Piped water	0.55	0.50
Clean toilet	0.75	0.43
Basic sanitation	0.37	0.48
Self-employed worker	0.22	0.41
Government worker	0.05	0.22
Private sector worker	0.35	0.48
Family worker	0.07	0.26
Primary industry	0.16	0.37
Secondary industry	0.16	0.37
Service industry	0.45	0.50
Safe fuel use	0.74	0.44
Observations	5563	

Table A.4: Association between health and circumstances

	(1) Underweight	(2) Overweight	(3) Hypertension	(4) Diabetes
Paternal BMI in childhood	-0.0136***	0.0204***	0.00364**	0.000689
Maternal BMI in childhood	-0.00835***	0.0228***	0.00728***	0.00193
Paternal height in childhood	-0.00100	0.000883	0.000368	0.000271
Maternal height in childhood	-0.00184**	-0.000165	-0.000119	0.000287
Paternal education years	0.00122	-0.00200	-0.00178	-0.00384
Maternal education years	0.00382**	-0.000461	0.00108	0.00256
ln(Wealth) in childhood	0.00123	-0.00187	-0.00310*	0.00396
Books 11-25	0.0242**	-0.0129	-0.0135	-0.0100
Books 26-100	0.00628	0.0163	0.0180	0.000210
Books 101-200	0.0215	0.00183	-0.0605	0.168**
Books 200-	-0.0336	0.0814	-0.0812	-0.0201
Had hunger	-0.0238	0.0339	-0.0153	-0.0148
Javanese	0.00417	-0.0194	-0.0150	0.0292
Male	0.0660***	-0.177***	0.0454***	-0.0201*
Islam	0.0477**	-0.00765	0.0168	0.0405
Hindu	0.0496	-0.0148	0.00740	0.151**
Catholic	0.0566	0.00429	0.00177	0.0269
Family size in childhood	0.00220	-0.00776**	0.00128	0.000679
Teenage pregnancy	-0.0170	0.0243	-0.00256	-0.00943
Mother married at age 12	-0.0129	-0.0351	0.0226	-0.0118
Biologicaal mother at age 12	-0.0309	0.0797**	0.0125	0.0549
Biologicaal father at age 12	0.0254	0.0124	-0.0179	-0.0512
Electricity	0.00555	0.0142	-0.0186	-0.00991
Clean water	0.0100	-0.0162	0.00362	-0.0119
Clean toilet	0.00959	-0.00298	0.0146	0.000877
Sanitation	0.0106	-0.0386*	0.00703	0.0141
Finished wall	-0.00776	-0.0271*	-0.0131	0.00609
Finished floor	0.0126	0.0288*	-0.00982	-0.0148
Finished roof	0.0209	-0.0325	0.0160	0.00737
Number of rooms	-0.00867***	0.00781**	0.00622**	0.000330
Father self-employed worker	0.0736	-0.104	0.0280	0.0129
Mother self-employed worker	0.110	-0.258	-0.0965	-0.00542
Mother goverment worker	0.0978	-0.142	0.0127	0.00159
Father goverment worker	0.0885	-0.269	-0.103	-0.0124
Mother private sector worker	0.0670	-0.0960	0.0387	0.0180
Father private sector worker	0.109	-0.246	-0.120	-0.0279
Mother family worker	0.141	-0.120	0.0369	-0.0157
Father family worker	0.114	-0.228	-0.101	0.0168
Mother primary industry	-0.0376	0.155	0.00827	0.0223
Father primary industry	-0.148	0.309*	0.100	-0.00153
Mother secondary industry	-0.122	0.300*	0.0897	0
Father secondary industry	-0.0216	0.154	-0.00330	0.0266
Mother service industry	-0.0398	0.159	-0.0106	0.000817
Father service industry	-0.126	0.286	0.104	0.0104
Knowledge of public hospitals	-0.0164*	0.0121	-0.00347	-0.0200
Knowledge of private hospitals	-0.00244	-0.00423	-0.0162	-0.00273
Knowledge of health centres	0.00153	-0.0227	-0.00313	-0.00117
Knowledge of private clinics	-0.00156	0.0134	0.0189	-0.00365
Knowledge of private physicians	-0.0158	0.0226	0.000168	0.0112
Knowledge of nurses/paramedics/midwife practitioners	0.0126	0.000261	0.00290	-0.00376
Urban in childhood	-0.00383	0.0461***	0.00969	0.00931
North Sumatra	-0.0130	-0.0520	-0.0325	0.0641
West Sumatra	0.0156	0.00815	-0.0385	0.0323
South Sumatra	0.00545	-0.0242	-0.0430	0.0903**
Lampung	-0.00811	-0.0667*	-0.0355	0.0539
West Java	0.0193	-0.0152	-0.000518	0.00501
Central Java	-0.00520	-0.0400	0.00441	-0.0000913
Yogyakarta	0.0196	-0.0165	-0.00630	-0.0103
East Java	0.00799	-0.0162	0.00290	0.0619
Bali	-0.0444	0.0305	-0.0458	-0.0679
West Nusa Tenggara	0.0535**	-0.142***	-0.0266	0.00824
South Kalimantan	0.0224	-0.0190	0.0508*	0.00240
South Sulawesi	0.0146	-0.0700*	-0.0377	0.144***
Constant	0.881***	-0.693***	-0.214	-0.230
Observations	5523	5523	5481	1148
R-squared	0.0585	0.119	0.0256	0.0789

Marginal effects are shown

* $p < 0.1$, ** $p < 0.05$, *** $p < 0.01$

Table A.5: Association between health and efforts

	(1) Underweight	(2) Overweight	(3) Hypertension	(4) Diabetes
Education years	0.0219***	-0.0318***	-0.00664	-0.00644
ln(wealth)	-0.0137	0.000288	-0.00164	0.0341*
ln(food expenditure)	-0.0479	0.205***	0.0145	0.0584
Prepated food ratio	0.0300	-0.184	0.0325	-0.121
Staple food ratio	-0.105	-0.584	-0.414	-0.443
Vigorous exercise	0.0157***	-0.0333***	-0.000733	0.00192
Moderate exercise	-0.0119***	0.0298***	0.00672	-0.00666
Instant noodle	0.0816***	-0.104***	0.00929	-0.00121
Fast food	-0.0856	0.171**	0.0919*	0.0456
Soft drink	-0.0572*	0.0523	-0.00900	-0.0276
Fried snacks	0.00553	0.0143	0.0130	-0.0240*
Sweet snacks	0.0236	-0.0337	-0.0412***	0.000941
Clean water	-0.0371	0.00607	0.0446	-0.0133
Clean toilet	-0.0386	0.0767	0.0298	-0.103
Sanitation	-0.0521	0.0976	0.00719	0.0214
Self-employed worker	-0.121	0.185*	0.169**	0.0570
Government worker	-0.106	0.125	0.140	-0.0635
Private sector worker	0.0287	0.0741	0.184**	-0.0391
Family worker	0.171*	0.130	-0.00381	0.264**
Private industry	0.0402	-0.297***	0.00115	-0.0902
Secondary industry	0.168*	-0.213	-0.122	0.120
Service industry	0.143*	-0.290**	-0.0710	-0.191*
Safe fuel use	-0.119***	0.130**	-0.0930**	0.0753
Constant	0.633	-1.732**	0.0130	-0.848
Observations	5504	5504	5462	1143
R-squared	0.0291	0.0685	0.0124	0.0310

Marginal effects are shown

* $p < 0.1$, ** $p < 0.05$, *** $p < 0.01$
